# May the force be with your (immune) cells: an introduction to traction force microscopy in Immunology

**DOI:** 10.3389/fimmu.2022.898558

**Published:** 2022-07-28

**Authors:** Farah Mustapha, Kheya Sengupta, Pierre-Henri Puech

**Affiliations:** ^1^ Laboratory Adhesion Inflammation (LAI), INSERM, CNRS, Aix Marseille University, Marseille, France; ^2^ Centre Interdisciplinaire de Nanoscience de Marseille (CINaM), CNRS, Aix Marseille University, Marseille, France; ^3^ Turing Center for Living Systems (CENTURI), Marseille, France

**Keywords:** traction force microscopy (TFM), immune cell, mechanics, force, mechanobiology

## Abstract

For more than a couple of decades now, “force” has been recognized as an important physical parameter that cells employ to adapt to their microenvironment. Whether it is externally applied, or internally generated, cells use force to modulate their various actions, from adhesion and migration to differentiation and immune function. T lymphocytes use such mechano-sensitivity to decipher signals when recognizing cognate antigens presented on the surface of antigen presenting cells (APCs), a critical process in the adaptive immune response. As such, many techniques have been developed and used to measure the forces felt/exerted by these small, solitary and extremely reactive cells to decipher their influence on diverse T cell functions, primarily activation. Here, we focus on traction force microscopy (TFM), in which a deformable substrate, coated with the appropriate molecules, acts as a force sensor on the cellular scale. This technique has recently become a center of interest for many groups in the “ImmunoBiophysics” community and, as a consequence, has been subjected to refinements for its application to immune cells. Here, we present an overview of TFM, the precautions and pitfalls, and the most recent developments in the context of T cell immunology.

## Introduction

The adult human body has approximately 10^13^ cells, and its fate, in terms of tissue and organ development and homeostasis, depends on how well these cells interact with one another and with their environment (see, for example (1–4) and references therein). A wealth of cell biology reports has documented the biochemical aspect of these interactions, identifying the networks of secreted ligands, cell surface receptors, intracellular signaling pathways, and transcriptional factors at play. However, as cells live in a physical world, the mechanical aspect of such interactions cannot be neglected. Indeed, the last few decades of research have confirmed that cells do sense the mechanical forces arising from their environment; they actively respond to them through mechanically driven biological actions, such as adhesion, migration, division, differentiation, and even apoptosis - a process termed mechanotransduction (4). Mechanotransduction appears to be present in almost all interactions between a given cell and its environment, including immune cells.

For T lymphocytes, the initiation of an adaptive immune response necessitates the interaction of naive T cells with antigen presenting cells (APCs). This interaction starts with the T cell receptor (TCR) recognizing an antigenic peptide presented on the major histocompatibility complex (pMHC) of the APC. Once the TCR binds to a cognate pMHC, the T cell can be seen applying cycles of pushing and pulling forces on the APCs. These forces, generated from the rapid reorganization of the T cell cytoskeleton upon activating stimuli, may participate in the formation of a specialized cell-cell interface termed the “Immunological Synapse” (IS), encompassing additional receptor-ligand pairs. Through these interactions, the APC relays a highly orchestrated series of signals that drive T cell activation, proliferation, and eventual differentiation (3).

In recent years, it has become increasingly clear that the mechanical forces generated at the IS are essential for the proper activation of T cells; several of the cell surface receptors participating in the IS are mechanosensitive proteins, and the forces originating from the constant remodeling of the cytoskeleton play an important role in regulating them (5–7). It has been also proposed that both the amplitude and the time evolution of the forces applied through the TCR contribute to rapid discrimination of the antigenic peptides (8). Moreover, there is evidence suggesting that T cells and APCs use mechanical forces as a form of communication to transmit information across the synapse (2).

**Figure 1:**
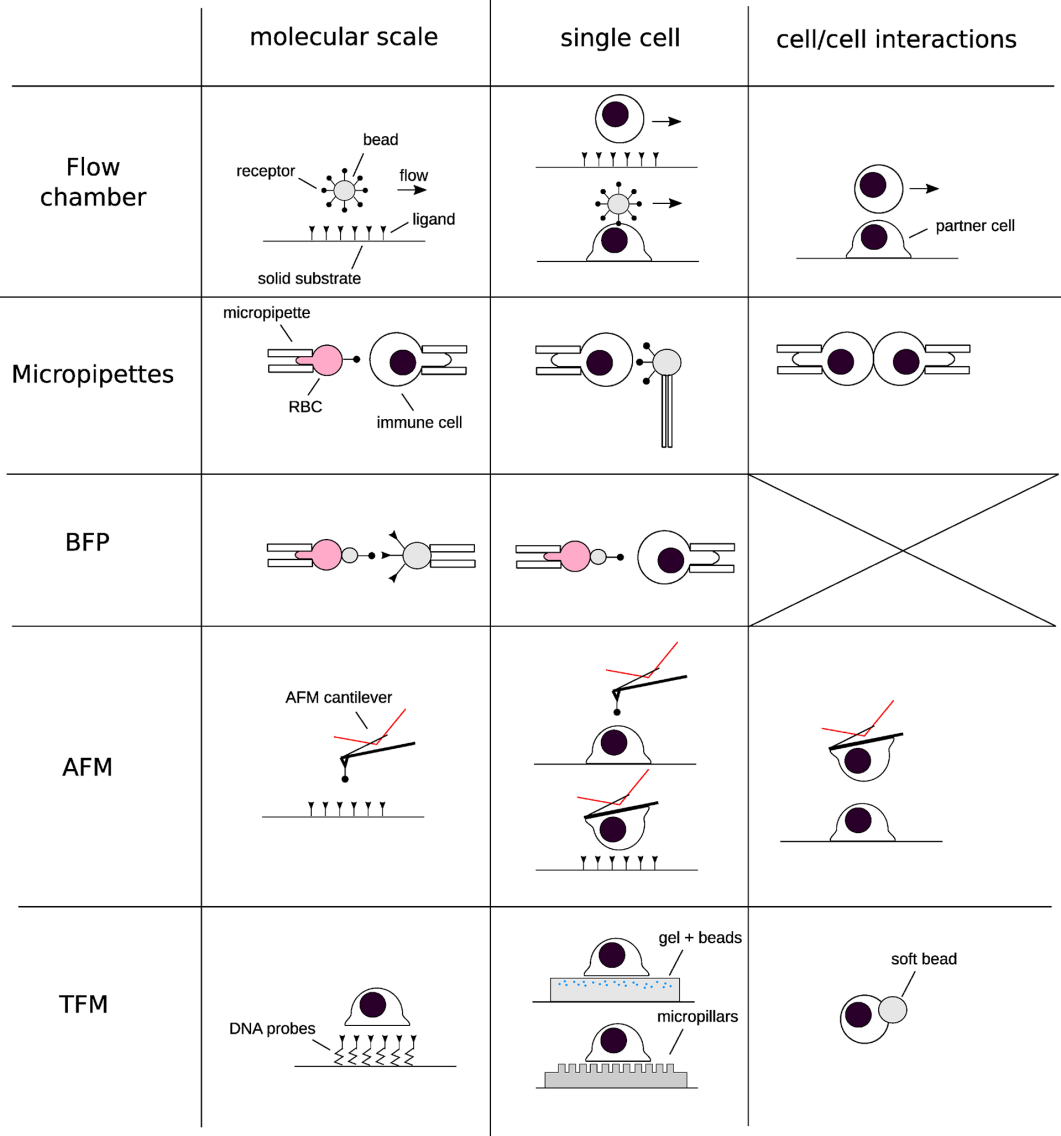
Schematics of the techniques that have been used for deciphering the implications of forces in immunology at different scales of space (from molecular to cell/cell interactions) and times. Each row represents a “group” of similar techniques, and each column a given spatial scale. A selection of references corresponding to each technique, *restricted to their application to immune cells:* Flow chamber: (10–12) Micropipettes: (13–16). Biomembrane Force Probe: (17–20). Atomic Force Microscopy: (21–26) Traction force microscopy and related techniques: (27–36).

Thus, given the substantial impact of mechanical forces on the behavior of T cells, and knowing that even comparatively moderate defects in T cell activation can lead to autoimmune diseases on one hand, and immunodeficiency on the other, it comes as no real surprise that elucidating the precise mechanisms underpinning mechanotransduction is of significant interest to researchers in the area of fundamental and applied immunology, and biophysics. Clearly, a knowledge of both the intracellular and extracellular forces is required. Owing to this demand, the last two decades have witnessed a burst in novel experimental methods that have been employed to quantify cellular forces (4, 8, 9). These include, but are not limited to, Atomic Force Microscopy (AFM), Optical Tweezers (OT), Bio-membrane Force Probes (BFP), and Traction Force Microscopy (TFM) (**Figure 1**, and references within the caption).

This review will focus on methods that are now collectively known as Traction Force Microscopy (TFM). TFM is essentially a technique that permits the quantification of cellular traction forces *via* the non-invasive optical imaging of deformations induced by the cell. Though the term was initially used to refer to the forces exerted by adherent cells on 2D linear elastic substrates (37), it has since been adapted for quantification of three dimensional (tangential and normal) forces exerted onto 2D, 2.5D and 3D substrates.

## Making invisible forces visible

Broadly speaking, forces are not an experimentally directly accessible quantity; they have to be inferred from the fact that they create some type of deformation or motion. The relation between deformation/motion and force is described by the classical laws of physics, one such example being Hooke’s law for the deformation of a linear elastic spring: F = k Δx, where F is the force, k is the spring constant and Δx is the extension of the spring. Without a measurement of Δx, no statement on F would be possible (k is a constant that can be obtained from a calibration experiment). In order to measure Δx, the relaxed reference state of the spring in the absence of any force has to be known.

Consequently, all measurements of cellular forces must start with the identification of a suitable strain gauge and incorporating it into a cell culture setup. One straightforward way of doing so is by replacing the traditional glass or plastic cell culture plates with a substrate capable of deforming under force. The earliest attempt at this was by Harris et al. who used a thin silicon rubber to show that fibroblasts generated elastic wrinkles when crawling (38). They named the force *“traction”*, comparing it to *“the traction an automobile’s wheel exerts on the highway surface”*. However, because wrinkling is an inherently non-linear and complex process, the forces couldn’t be accurately quantified.

## Continuous versus discrete anchoring

**Figure 2:**
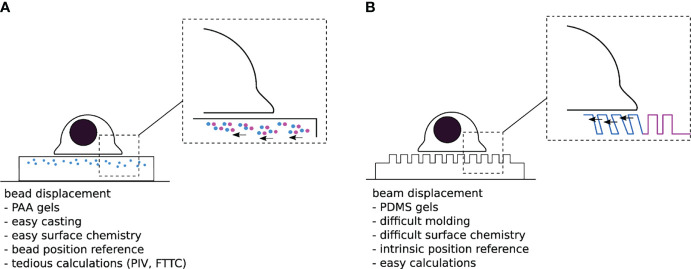
**(A)** “Traditional” TFM with PAA gels doped with sub-resolution fluorescent beads. One difficulty is to assess the non-perturbed bead position either at the beginning or the end of the experiment. **(B)** Micropillar based TFM. The typical size of a pillar is 1µm diameter over 10µm length, for a 1 µm interpillar distance, and a hexagonal compact 2D distribution-the numbers given here are typical orders of magnitude for these parameters). The unmolding step in the substrate fabrication process can be quite delicate, while the force localization and calculation are rather trivial.

Despite this seminal experiment remaining a rather qualitative observation, it inspired the design and development of alternative systems capable of quantitatively measuring traction forces. Nearly two decades later, in 1999, Dembo and Wang officially introduced “*Traction Force Microscopy”* – TFM - as a method to quantify forces exerted by adherent cells on compliant substrates (37). They replaced the silicon membranes with thicker, linearly elastic, hydrogels and adopted fluorescent beads as fiducial markers, instead of relying on wrinkles to report substrate deformation (**Figure 2A**). Above all, these changes replaced the generally nonlinear and mathematically complex description of wrinkle formation with a classical, linear, continuum mechanics model from material science (39, 40), thus opening the way for systematic force measurement.

In an attempt to further simplify the computationally intensive force calculations required for continuous hydrogels, Tan and colleagues introduced an elegant alternative system for TFM in 2003 (41). Theirs consisted of cylindrical polymeric pillar arrays, fabricated by soft lithography, where cellular forces can be laterally decoupled in a series of local strain gauges; once cells adhere to the protein-coated pillar tops, they bend them away from their unloaded position. By estimating this deformation and applying the classical beam bending theory, one can then calculate the local traction forces exerted by the cells (**Figure 2B**) (41, 42). Despite the obvious advantage of using such discrete adhesive surfaces (i.e., load-free reference position is readily available and the deflection of a given pillar only depends on the force applied to that particular pillar), the pillars themselves represent a major flaw in the system: They impose arbitrary restrictions on the size, shape, and location of cellular adhesions, and consequently control where and how cells transmit force (43, 44). In addition, if the cell makes adhesive protrusions that extend into the substrate beyond the very top of the pillars, the classical calculation is not applicable. Thus, even though forces can be elegantly calculated using such a system, it remains unclear how these calculations relate to those actually transmitted in the native cellular environment.

Though the pillar arrays system suffered from several intrinsic limitations, it is crucial to highlight that the concept behind it served as a foundation to build a number of new approaches that translated the “reference free” and “computationally easy” force reconstruction onto flat 2D TFM substrates. These include the micro-patterning of cell adhesive islands (45, 46), the lithographic photoresist of ordered arrays (47, 48), as well as the nano-patterning of quantum dots (QDs) on linearly elastic substrates (49). Using these technologies, a regularized grid of reporter structures allows the determination of deformation of continuous 2D substrates without the need of a reference frame. However, as these patterns may represent the only sites where cells can exert force, similar to the pillars, the artificial constraint on cell force location will impact the physiological relevance.

## From 2D to 3D TFM

Whether it’s the continuous hydrogels from Dembo and Wang, or the pillar arrays from Tan and colleagues, both systems were originally developed with the aim of quantifying forces generated by *adherent* cells on 2D substrates. This was based on the assumption that cellular forces are predominantly tangential (in-plane, x, y), and that the forces normal to the substrate (out-of-plane, z) are negligible (50). However, since then, it has become evident that cells interacting with adherent substrates exert forces in 3D, and that the out-of-plane traction components are often comparable to the tangential ones (51, 52).

To account for these realizations, classical 2D TFM has been extended to 2.5D and 3D TFM (53–56).

2.5D TFM refers to the measurement of tangential and normal cellular forces exerted onto 2D substrates, not to be confused with “true 3D” TFM that quantifies forces exerted in 3D space (substrate). Nevertheless, in either case, by obtaining both the in- and out- of plane displacement fields of fiducial markers (e.g., fluorescent beads or patterns) using high-resolution image processing, for example through z-stack or astigmatic imaging (56), one can then reconstruct the “3D” force fields exerted by the cells.

While resolving normal traction forces is in itself difficult, given that it requires significant computational power, in addition to an appropriate imaging modality (discussed below), 3D TFM in specific comes with its unique set of challenges. Typically, in 3D TFM, cells (e.g., fibroblasts) are encapsulated *within* a deformable 3D extracellular matrix (ECM) scaffold material (e.g., collagen or fibrin fibers), pre-loaded with fluorescent beads (57). Unlike in 2D and 2.5D TFM, where the synthetic substrates can be fully characterized, biopolymers such as ECM materials are mechanically complex (57); they are constantly being synthesized, degraded and remodeled by cells. It is thus difficult to discern whether the recorded deformations are caused by one of those processes or by actual cellular forces. Besides, natural ECM is composed of fibers with highly non-linear force-extension relationships, meaning extracting traction forces from deformations is not possible using classical mechanics approaches. An innovative solution around these difficulties was put forth by Legant et al. who performed 3D-TFM with polyethylene glycol (PEG) hydrogels, incorporating domains that allowed for both adhesion (fibronectin RGD binding domain) and degradation (matrix metalloproteinase susceptible linkers) by the embedded cells (58). It is important to note that 3D TFM is not quite physiologically relevant when studying lymphocytes, potentially more so for other immune cells such as macrophages.

Another noteworthy innovative TFM adaptation involves the use of deformable hydrogel microparticles for force quantification (35). Though this approach does not follow the classical definition of either 2.5D or 3D TFM, since neither the substrates are 2D, nor are the cells encapsulated, it does allow the quantification of tangential and normal forces applied to a sphere of adjustable size, and can therefore be quite intriguing for the investigation of cell-cell interactions. For example, studying T cell-APC and cytotoxic T cell-infected cell interactions where membrane tension is essential for immune synapse stabilization (59) and perforin (a hydrophobic protein that forms pores in the target cell membrane) secretion (60).

## Making the right material choices

**Figure 3:**
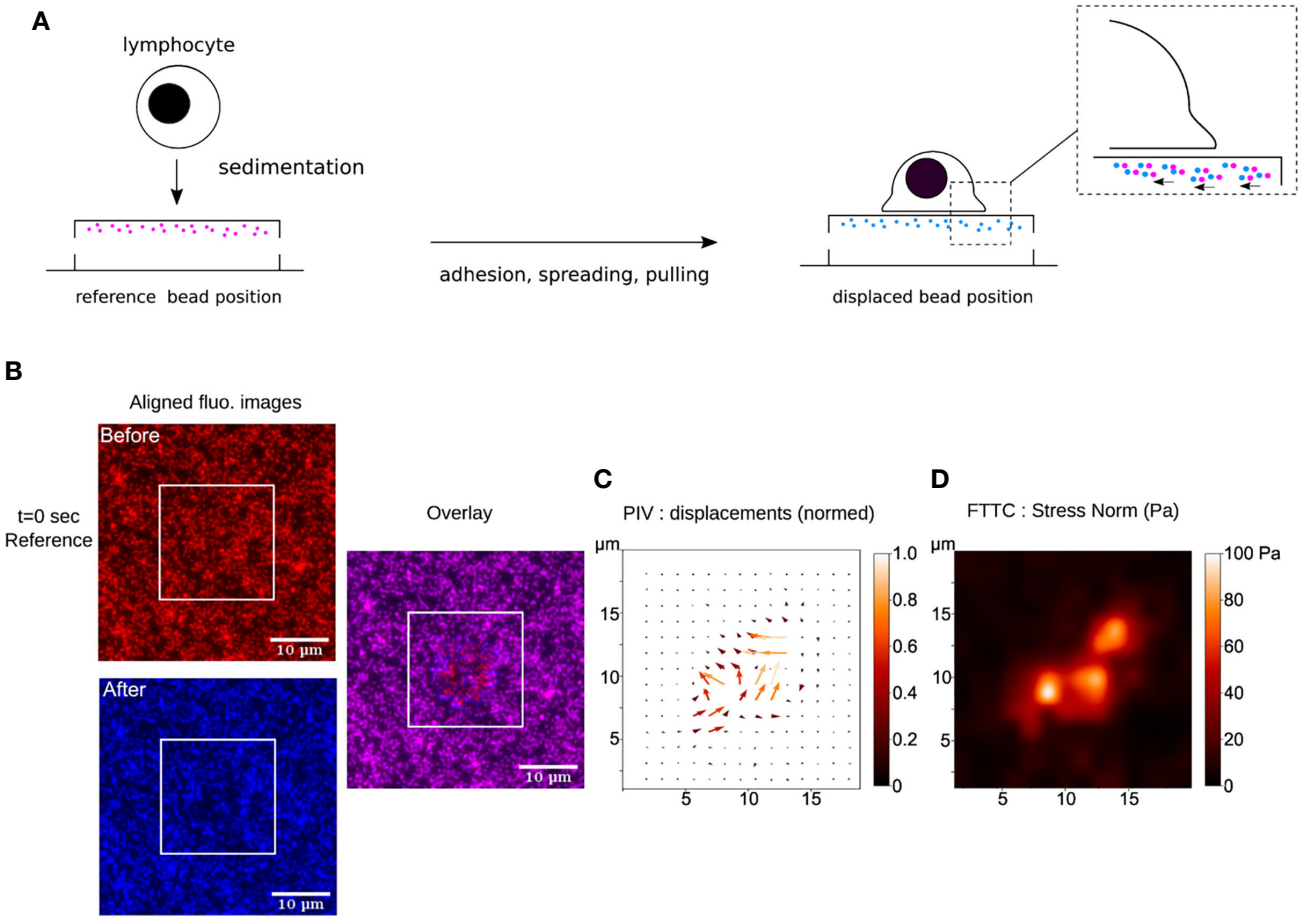
**(A)** Schematics of TFM for the study of early interactions of a primary human T lymphocyte with an ultra-soft APC-mimicking PAG doped with fluorescent nanobeads. **(B)** Raw fluorescence images, before and after the cell has landed, aligned to remove sample lateral drift. These ROIs are cut from original large field epifluorescence movies. The white squares indicate where a T lymphocyte has landed, as observed in bright-field transmission microscopy (not shown). The overlay shows the displacement of the beads due to cellular force. **(C)** Result of PIV calculation (over the zone delimited by the white square, where the cell sits) showing the constructed vector map of bead displacement field, taking t=0 sec frame (before the cell has landed) as the cell-/stress-free reference. The displacements have been normalized. **(D)** Result of FTTC calculation showing the gaussian smoothed map of stress norm. The data presented here has been processed using open-source softwares (Fiji/ImageJ (61), Python), following (29).

Despite the many exciting developments in the broad field of TFM, the most commonly used system to measure cellular traction forces remains the one designed by Dembo and Wang in 1999: TFM on continuous and linearly elastic substrates embedded with fluorescent beads (37) (**Figure 3**). The most popular substrates used in this system are polyacrylamide gels (PAGs) and polydimethylsiloxane elastomers (PDMS, also called silicone). However, two unique features have given PAGs an edge over their counterparts. First, PAGs span an excellent range of elasticities (62). By simply varying the concentrations of acrylamide and N,N′-methylenebisacrylamide-the building blocks of PAGs- while retaining the same surface chemistry, the stiffness of the PAG can be adjusted to mimic that of most biological tissues (typically from 100 Pa to 100 kPa). Second, PAGs are generally non-fouling, meaning they are nearly inert as adhesive substrates. The same chemical stability and non-adherence that allows the usage of PAGs for the electrophoretic separation of nucleic acids and proteins, also guarantees that neither cell surface receptors nor adhesive proteins present in the serum can bind directly to the gel. Consequently, only molecules *covalently* grafted on the gel surface can act as ligands for the cells (29). In comparison, different formulations of PDMS are required for it to span a similar range [1 KPa- 1 MPa, ‘Q-gel’ is the more suitable choice for low elasticities and ‘Sylgard’ for the high ones; (63)]. Additionally, being extremely hydrophobic, PDMS requires supplementary passivation to prevent the non-specific adsorption of proteins onto its surface.

It is generally accepted that the experimental setup used for TFM has a great influence on the achievable result, both in accuracy and quality. Thus, regardless of the chosen material, a number of key considerations must be taken into account when designing a TFM substrate.

First, the thickness of the substrate needs to be sufficient. “Cells may not see or hear’’, but they can certainly “feel” their surroundings and sense a collective stiffness. Just like the princess in Hans Anderson’s fairy tale who felt a small pea beneath a stack of soft mattresses, cells too can feel the stiffness of a rigid support buried beneath a soft layer, even if they’re not in direct contact with it. The soft layer, in this case the substrate, must be sufficiently thick such that the cells feel and respond to its softness rather than the rigidity of the underlying glass.

Second, the stiffness of the substrate must be tuned to fit the biological system under investigation. Different cell types exert forces over a wide range, and thus the chosen stiffness must be able to manifest the exerted forces as an appropriate deformation. On one hand, if the substrate is too stiff, the cells will not be capable of effectively deforming it, resulting in insufficient bead displacement, and rendering the calculation of force impossible. On the other hand, if the substrate is too soft, then the bead displacement may be too large, thus breaching the linear-response regime and making the linear-elastic theory inapplicable. A starting point for cells hitherto unexplored in terms of force measurements, is to consider the elasticity data reported for cells or tissues that the cells under consideration interact with. For example, when working on T cells, a stiffness such as the one reported for antigen presenting cells may be the appropriate starting choice (64).

Third, considerations of roughness and porosity are important. Given the cross-linked nature of PAA and PDMS, their stiffness is related to the mesh size of their molecular polymer network; the stiffer they are, the smaller the mesh size. Thus, an additional restriction would be that the substrate must be stiff enough to grant the formation of a sufficiently small mesh size capable of trapping the beads inside of it. It is important to note that mesh size may also influence the surface density of the functionalized proteins (65).

Fourth, the density of the fiducial markers needs to be optimal. Bead density in the substrate, of course in conjunction with the optical technique chosen for observation, directly determines the accuracy of force recovery (61). Thus, it must be carefully chosen in accordance with the spatial scale, the magnitude of the forces being measured, and the image analysis method to be used later. The density of the beads must be high enough to capture the spatial intricacies of the traction force field. If the bead density is too low, then in certain areas the deformation may go unreported and thus the traction information may be incomplete. Alternatively, if the bead density is too high, the image of the beads may overlap and nearby beads may not be resolved, thus concealing details of their relative displacement.

## Quantifying displacements

The fundamental principle behind TFM has remained the same since its conception: when cells adhere or migrate over sufficiently compliant substrates, they exert traction forces that can deform said substrate. These deformations are spatially and temporally mapped by monitoring the changes in lateral position of sub-resolution fluorescent beads embedded just below the cell accessible, functionalized surface.

In order to measure cell traction forces, (at least) two images of the substrate have to be acquired: One image of the bead field while the substrate is subjected to cellular forces (i.e., the stressed state) and another image of the bead field in the absence of cellular forces (i.e., the relaxed state). The image of the beads in their relaxed state can either be obtained *before* cell engagement (28, 66) or *after* cell detachment using EDTA or cocktails of proteolytic enzymes such as Trypsin or Accutase (67). Provided that the substrate is linearly elastic, the beads should return back to their relaxed state once the cells, and therefore the exerted forces, have been removed. The displacement caused by the cells can then be computed by comparing the bead positions in the stressed state to that in the relaxed state.

There are currently two main approaches to perform this comparison, either by localizing and tracking each individual bead, also known as single-particle tracking (SPT, (68, 69)), or by correlating displacements with regions of an image, also known as particle image velocimetry (PIV, (70)).

SPT identifies and tracks individual bead centroids by utilizing single particle localization algorithms. Basically, these algorithms scan all the pixels in the relaxed image to identify the pixel coordinates of the fluorescent beads (referred to as pixel intensity maxima). For each bead that is tracked, a box of pixels centered around the maximum intensity pixel is designated. The relative pixel intensities in that box serve as a ‘‘fingerprint’’ for the tracked bead, which is then used to find the coordinates of the corresponding ‘‘fingerprint’’ in the stressed image. This process is repeated for every bead in the image. Usually such procedures are able to track the bead displacements with submicron resolution (71).

Alternatively, one can forgo identifying and tracking individual bead centroids, and instead use PIV to calculate and project displacements on a grid, using image cross-correlation. To do that, both rest and stressed images have to be first partitioned into small interrogation windows. The pattern of an interrogation window in the first image is correlated with a region of equal size in the second image that is shifted pixel-wise in the vicinity of the location of the interrogation window of the first image. The result of this operation is a local correlation map of a specific bead pattern. The position of the maximum correlation value within this map is the most probable displacement of the bead pattern of this specific interrogation window.

Because PIV requires the image to be divided into smaller regions, some of the displacement occurring in the sub-regions might be lost. To minimize this loss, the selection of a “correct” PIV window is critical. If the window is too large, fine detail regarding the bead displacement will be lost, and the overall resolution of the force will be compromised. Alternatively, if the window is too small, such that it contains no distinguishable features (eg. a too small number of beads or even no beads to the extreme limit), the correlation between frames will be unreliable and prone to error due to the creation of non-existing displacement. Such a choice is influenced by the density of beads but also by the scale of the features one expects to record.

Both of these approaches have their own limitations, and they can also be combined (30, 69). SPT potentially yields higher accuracy but may introduce incorrect bead matches between the relaxed and stressed images which contaminate the true displacement data. PIV on the other hand is robust against mismatches as well as sample drift in the z direction, however, it is doubtful to obtain comparable lateral accuracy and resolution. Nevertheless, they have both been utilized in 2D TFM with minimal modifications. The question of which approach to use depends on the expected nature of the forces. For example, in the case of focal adhesion forming cells where forces are likely to result in a collective motion of a group of beads, it would probably be more appropriate and practical to use an approach that depends on image cross correlation instead of individual bead displacement.

## Mapping forces

The final step in TFM is to convert displacements into a map of cellular traction stresses or forces. In other words, a relationship, derived from the physics of materials, is needed to describe the deformation of a material in response to a force applied onto its surface. Although this conversion fundamentally requires solving a stress-strain problem, several approaches have been developed to do so, the two main ones being the forward approach and the inverse approach.

The forward approach is more straightforward and computationally efficient. As the name suggests, the stress tensor is calculated directly from a three-dimensional displacement field, using the constitutive law of the material, and the surface traction is calculated from the 3D stress field (72). One major advantage for this method is that it can be easily applied to nonlinear, viscoelastic, or other material constitutive properties without having to modify the general mathematical framework. Nevertheless, there are two major trade-offs to using it. First, a 3D or quasi 3D displacement field is needed and second, noise effects may become very important. In traditional 2D TFM where fluorescent beads are embedded in the substrate, the stress field is not known immediately at the cell-substrate interface, it’s rather measured at the layer of beads closest to the interface. Consequently, in order to calculate the forces experienced at the true substrate interface, some method of extrapolation must be implemented to estimate the stress field at the interphase from that at bead level. This estimation might introduce significant error if one can’t ensure that a large enough number of beads is present quite near to the substrate surface.

One way to address this concern is to adopt the inverse approach. In this approach, the traction field becomes a convolution of the displacement field and Green’s function. It’s important to note that the utilization of Green’s function imposes several key assumptions. First, forces are mainly exerted along the substrate surface rather than normal to it. Second, the substrate is estimated to be a 2D elastic plane extending laterally to infinity (a semi-infinite half space). Third, the strains are small and thus the substrate deforms under a linear elastic regime. Lastly, the substrate material remains homogeneous in both relaxed and stressed states. Even if all these assumptions are experimentally met, the inverse approach still suffers from two major limitations. First, upon inversion, the calculated forces become very sensitive to high frequency fluctuations (i.e., noise) in the displacement data. To solve this problem, a pre-smoothing, also known as regularization, must be implemented to obtain a reasonable solution (73)). The regularization coefficient must be carefully chosen so as to provide a balance between how well the solution fits the noise-distorted experimental displacement data and the overall magnitude of the traction forces. If the solution is over-regularized, the data will become over-smoothed and the resolution of the recovered forces will be lost. Alternatively, if the data is under-regularized, the solution will overfit the noise in the displacement and will thus be a false representative of the traction forces. Secondly, the computation needed to solve the inverse problem and implement the additional regularization steps is quite time-consuming and computationally expensive. The most common and general way to solve this problem is by using Fourier Transform Traction Cytometry (FTTC) whereby, essentially the integrated displacements are transformed into Fourier Space and the calculations are performed using matrix multiplications.

## Improving detection in 2D (and 3D)

The accuracy and resolution of TFM ultimately depend on the spatio-temporal resolution of the optical microscopy technique with which it is accompanied. The spatial resolution is a limit imposed by the resulting finite size of the point spread function (PSF) associated with each fluorescent bead. At high densities, the PSF of the beads begin to overlap, hindering the reliable tracking of their displacement. Similarly, the temporal resolution also influences the ability to reference and track individual beads over time. Not to mention that at low time resolutions, dynamic processes are concealed, whereas at higher time resolution, requiring more frequent imaging, phototoxicity as well as photobleaching become a concern. As such, experimentalists often find themselves forced into a trade-off between spatial and temporal resolution.

The first straightforward attempt to partially overcome these limitations came from Sabass et al. who proposed to incorporate fluorescent beads of two different colors to increase the allowed bead density while decreasing the noise and irregularities in bead tracking (69, 74). To further improve the spatial resolution of TFM from the micron to the submicron scale, Colin-York et al. combined super-resolution stimulated emission depletion (STED) microscopy with TFM (2D STED-TFM) (75). STED-TFM allowed a 5-fold improvement in the resolution of the tracked bead displacement field, yielding a much finer recovery of force compared to standard laser scanning confocal microscopy. This step forward however, came at the expense of increasing the image acquisition time to a few minutes for each field of view due to the STED scanning. Additionally, the high laser intensity required for fluorescence depletion diminished the biocompatibility of this approach. The same group later addressed these problems by developing live-cell super-resolution 3D SIM-TFM, a technique combining structured illumination microscopy (SIM) and TFM. Because SIM is a wide-field technique, it does not rely on image raster scanning, and thus, unlike STED, allowed faster acquisition times (11 ms per frame, 15 frames per super-resolution image in 3D mode), and at a significantly lower fluorescence excitation light, thus increasing the number of images that can be a acquired at a given time frame while minimizing the effects of photobleaching (76, 77).

To overcome the need for the axial scanning required for the 3D imagining of the beads using 3D SIM-TFM, and further increase the speed of acquisition, they later combined TFM with 2.5D astigmatic imaging (aTFM) and SIM in total internal reflection fluorescence microscopy mode (TIRF-SIM) (56). Astigmatic imaging allowed the 3D information in the ~1 μm zone surrounding the focal plane to be inferred from a single wide-field image, rather than having to perform multi-frame z-stack acquisitions, thus increasing image acquisition to up to 90 ms per SIM image frame, while the use of TIRF reduced the contribution of out-of-plane fluorescence and enhanced the overall quality (contrast) of the images. In their most recent work, they extended TIRF-SIM to 2D (2D TIRF-SIM-TFM), demonstrating a >2 fold increase in spatial resolution and >10 fold increase in temporal resolution in comparison to traditional TFM (78).

There are two main limitations that appear when using 3D SIM-TFM and TIRF-SIM-TFM (2.5D and 2D). Firstly, they necessitate the use of high numerical aperture objectives with narrow working distances which consequently diminishes the imaging depth and limits the thickness of the substrate that can be used. This is particularly problematic in TFM since, as mentioned previously, the substrate must be sufficiently thick to eliminate any mechanical influence coming from the underlying glass. Secondly, imaging the substrate-cell interface requires that the substrate has a refractive index similar to that of glass, such as PDMS (variety Qgel for example, (63)), which in itself comes with its own set of constraints, primarily the limited elasticity range that can be achieved. PAGs, having a refractive index similar to that of water, are therefore not directly amenable to such refined techniques.

It is also noteworthy that the availability of such advanced microscopy systems is likely limited by prohibitively high costs, either on the material side or on the development time needed to set them up, which often limits experimentalists to more classical fluorescence (epi or confocal) and phase-contrast microscopy. Normally, this would rule out the possibility of recovering 3D traction forces since traditional 2D imaging systems suffer from a relatively high degree of out-of-focus light-scattering. However, Hazlett et al. found an interesting strategy to get around that difficulty (54). They embedded a single dense layer of fluorescent beads on the PAG surface, and then obtained volumetric images of the beads by deconvolving the experimental epifluorescence images acquired using the PSF collected from a single bead in the images. Using SPT, they managed to quantify 3D volumetric bead displacements, and consequently, 3D stress fields.

## Insights into T cell biology from TFM data

In this section, we present a few prominent examples of recent insights gained into the workings of T cells thanks to TFM studies.

One of the earliest experiments implementing TFM for T cell studies examined the complementary roles of CD3 (parts of the TCR complex, responsible for signal transmission across the membrane) and CD28 (a costimulatory molecule participating T cell activation) in mechanosensing during primary human CD4+ T cell activation. Using PDMS pillar arrays presenting activating antibodies against CD3/or pMHCs and/or CD28, Bashour et al. confirmed that antigen recognition does in fact involve force exertion. They recorded traction forces of around 100 pN, exerted specifically through the TCR-CD3 complex, and which could be augmented with co-stimulation through the engagement of PI3K signaling pathways (27).

To examine the interplay between activation and adhesion, Tabdanov et al. also employed PDMS pillar arrays, but functionalized with activating anti-CD3 antibody +/- ICAM-1 instead (79). Their experiments showed that the incorporation of ICAM-1 significantly increased the cellular contractile stresses exerted by Jurkat T cells, in comparison to those recorded when only the TCR/CD3 complex was engaged. Combined with their experiments on micropatterned surfaces, their work highlighted a mechanical cooperation between the TCR/CD3 and LFA-1-ICAM-1 systems, whereby actin nucleation (governed by Arp2/3) downstream of TCR signaling sustained the growth of the LFA-1 dependent actin network, which in turn then provided the cytoskeletal tension to allow mechanical sensing, T-cell spreading and enhanced TCR activation.

Focusing further on the influence of the T cell cytoskeleton, Hui et al. used enhanced green fluorescent protein (eGFP)–actin expressing Jurkat T cells and poly-L-lysine-anti-CD3-coated polyacrylamide gels, to demonstrate the contribution of actin polymerization and myosin contractility in force generation and maintenance during T cell activation (28). With this system, they recorded peak stresses reaching 20–30 Pa and a total force of a few nanonewtons and showed that the EGFP-actin Jurkat T cells exerted larger forces on polyacrylamide gels of increased stiffness. This came in contrast to Bashour et al.’s work (27), where no change in traction force per pillar as a function of pillar stiffness was observed. Building on these results, the same group later utilized the same system to showcase the role of dynamic microtubules in regulating force generation at the T cell-substrate interface, through suppressing Rho contractility and actin flow (80).

Another study highlighting the role of actin in T cell force generation, was that of Savinko et al. (81) employing silicone-based gel substrates coated solely with ICAM-1. In these experiments, knocking out the actin-binding protein filamin A dropped the traction stresses exerted by mouse CD4+ effector T cells by approximately 50% (from ≃ 50 Pa to ≃ 25 Pa).

Linking cytoskeletal forces and effector function, Tamazalit et al. used PDMS pillar arrays presenting cognate p-MHC-I to show that CD8+ T lymphocytes employ F-actin rich protrusions, generated by Wiskott-Aldrich Syndrome protein (WASP) and the Arp2/3 actin nucleation complex, for synaptic force exertion and cytotoxic function (perforin and granzyme release; granzymes are proteases that induce cell apoptosis) - A process they termed as “mechano-potentiation” (82).

In an innovative approach, Vorselen et al. studied the interaction of eGFP-actin expressing cytotoxic T lymphocytes (CTLs) with activating (quantified as Ca^2+^ influx) soft (~ 300 Pa) deformable polyacrylamide microparticles (DAAM-particles, ≃ 15 µm in diameter) functionalized with cognate pMHCs and ICAM-1. Interestingly, using this technique, the shear stresses (~ 100 Pa) detected in the contact area (8 µm in diameter) between the CTLs and the microparticles were directed outwards, and as time progressed, localized indentations i.e., normal traction forces (up to 200 Pa, 0.5 nN total force) started forming within that area (35).

Similarly, Aramesh et al. also adopted an unconventional strategy to study both tangential and normal forces generated by T cells. Instead of using microparticles-doped gels however, they performed a functionalized bead assay whereby anti-CD3 and/or anti-CD28-functionalized 200 nm neutravidin-conjugated beads were bound onto the surface of biotinylated poly(ethylene glycol)diacrylate (PEGDA) gels or PDMS-based QGel, and Jurkat T cells were left to interact with them (55). In accordance with Bashour et al.’s observations (27), their experiments also showed that co-stimulation by CD28 does in fact enhance T cell forces, reaching up to 10 nN forces, and not surprisingly, the increased force generated correlated with increased Ca^2+^ influx, i.e., increased activation. However, what was truly intriguing about their data was that single T cell microvilli were targeting single beads, and within that T cell-microvilli contact, actin was forming a vortex-like ring structure where the TCR was enriched and CD45 was excluded. This comes in line with previous reports suggesting that the size-mediated exclusion of CD45 from the IS shifts the ITAM phosphorylation−dephosphorylation balance, thereby triggering TCR signaling (83).

Though this section has focused on the existing literature regarding T lymphocytes studies using TFM, several other immune cell types have been investigated using the same methodologies, often though to a lesser extent. These include neutrophils (31), B cells (30, 66), dendritic cells (84) and macrophages (36).

## Molecular sensors for force measurements

All the techniques mentioned thus far represent macroscopically large strain gauges that measure force maps generated by the cell, at the (sub)cellular scale. The same principle can be implemented at the nanoscale to measure the force borne by a specific molecule, through the interaction of a single receptor and ligand: provided that mechanical properties can be evaluated at the molecular scale, deformations of individual molecules, such as the extension of protein domains or DNA molecules, can be converted into forces. To this end, a great deal of effort has been dedicated over the last decade into the development of molecular force sensors (32, 85–87). These may not formally qualify as TFM but are included here because of their immense importance and potential.

The principal components of these sensors are deformable molecules that are sensitive to molecular tension, and that are labeled with a dye, a dye–quencher pair, or a dye-dye pair. Once force is applied onto the construct, its configuration will change, and consequently the fluorescent activity of the sensor will change as well. Thus, the experienced molecular force will eventually be reported as surface fluorescence loss, fluorescence gain, or Förster resonance energy transfer (FRET) efficiency change (88). Note that, similar to the substrates used in classical TFM, the responsivity range of the deformable molecule should match the range of the molecular force transmitted by the molecule under investigation.

While most common molecular force sensors are coated onto surfaces and are thus used to report the forces applied by cells onto said surfaces, another type can be used to measure forces *inside* cells. Typically, these constitute mechanosensitive proteins that have been engineered with fluorophore pairs and expressed in living cells; As they experience force, the separation distance between the fluorophores, and consequently the FRET efficiency, is altered, allowing for the real time measurement of intracellular forces across single molecules (89, 90).

Although such sensors provide an immediate readout of molecular forces, for several reasons, interpreting the obtained signals might not be as straightforward. First the effective spring constant of the elastic linker might depend on the local environment in the cell, even if previously calibrated by single-molecule force spectroscopy experiments (by Atomic Force Microscopy, Optical or magnetic Tweezers (91). Second, the fluorescent signal is a sensitive function of domain separation and relative orientation, thus, a direct conversion into force can be problematic (92, 93). Third, it is difficult to control the number of engaged sensors, consequently, the fluorescent signal cannot easily be integrated over a larger region. Not to mention that using such a technique allows the recovery of only the norm of the force exerted and not the exact direction of said force. Therefore, advanced molecular force sensors can be expected to complement, but not fully replace, traditional TFM in the future.

## Conclusions and perspectives

The last two decades have witnessed an upsurge in the development of a wide variety of techniques for probing cell generated forces. Though they have not been discussed in this review, they have been described in great detail elsewhere (See for example, for immune cells, (2, 8)). Despite their growing availability, such advanced biophysical techniques still require specialized skills and often expensive tools that are still far from becoming routine laboratory equipment in biology labs, unlike conventional molecular biology tools for examining gene expression and protein concentration. Perhaps the simplest of these techniques, and the one that is rapidly leading its way towards standardization, is TFM. Most likely, TFM has gained such wide adoption by the mechanobiology community because of its ease of implementation and longstanding history.

However, if we disregard for a brief moment its attractive simplicity, we will see that TFM suffers from very serious caveats. Primarily, the computational analysis required for tracking displacements and recovering force maps is quite complex, nuanced and difficult to validate. Even marginal errors in retrieving bead displacement will introduce large errors into the final stress and force fields. Moreover, as explained above, extracting force fields from displacement fields is a mathematically ill-posed problem that will introduce noise into the final measurements, and will thus necessitate regularization. Since there is not a “standard” regularization factor, which is quite logical since this value will depend on several experimental and numerical parameters, which are not uniform (e.g., bead size, bead density, substrate stiffness, cellular forces, and imaging parameters, methodology for calculating the beads displacements …), one could end up with either over-smoothed, or alternatively, under-regularized data, which does not faithfully represent the exerted cellular traction forces. Given such variable experimental and analysis protocols, comparing experimental values obtained in different laboratories becomes very difficult, especially, as is the case for any quantification of living systems, since biological diversity, such as cell culture conditions and cell passage number, may also impact the scatter in measured values.

Potentially, the only way to overcome these challenges is by utilizing reproducible and accessible standardized protocols, as well as implementing open source softwares for data analysis. Several startup companies that sell prefabricated substrates exist today, which is a partial step towards standardization - though in our experience their rigidities need to be verified by the end user. Python, ImageJ/Fiji, and even Matlab scripts are now available online for calculating stresses, force maps and energies from bead images (see for example https://sites.google.com/site/qingzongtseng/tfm, https://github.com/topics/traction-force-microscopy, https://github.com/MBPPlab/TFM_v1). Though this does not completely solve the problem, it is a step in the right direction towards standardization.

To further complexify the picture, the generation of mechanical forces by biological systems are space and time scales dependent, from cells, down to single molecules and up to entire organisms, lasting less than a few seconds up to hours and even over their whole lifetime. For example, looking at T cell activation, certain processes such as actin turn-over occur at the order of seconds, while others may take several minutes, such as the building of the IS, or more. Another important point is that, *in-vivo*, cells are interacting with different substrates/other cells and are constantly integrating the myriad of biochemical and physical signals rising from their microenvironment. Trying to recapitulate such intricate physiological conditions is extremely challenging, and so it remains difficult to understand how forces measured *in-vitro*, on mechanically simplified substrates, relate to those existing in living tissues or organs. A prominent example in T cell studies is that every interaction of a T cell with APCs will be made under different mechanical conditions as pointed out by Bufi et al. (64) leading to adaptation in experimental parameters, such as the substrate rigidity in TFM to accommodate for a precise encounter to be studied.

Therefore, before opting for one technique or the other, an investigator needs to make several critical decisions: (1) *in-vivo* or *in-vitro* (2) 2D, 2.5D or 3D, (3) spatial resolution- nanoscale or microscale- and/or temporal resolution-sub second, second, or minutes, (4) molecular scale forces or cellular scale forces. Another key point is deciding whether one time point quantification, and thus one force value, will suffice, or whether the process is dynamic and will require time-lapse measurements. We have specifically highlighted this point in our recent work using TFM on ultra-soft PAGs which showed that T cells exhibit distinct dynamic stress and energy patterns (29).

With the pace at which the field of mechanobiology is growing, it is not unreasonable to imagine that the next-generation tools for quantifying cellular forces will exhibit an extended range of measurable forces, an improved spatio-temporal resolution, and will re-create a more complex cellular microenvironment that will allow cells to experience a dynamically changing set of biochemical and physical conditions, more representative of that occurring in *in-vivo* settings. Though this may sound quite alluring, one has to keep in mind that the more complex our questions and experiments become, the more difficult it will be to extract meaningful correlations and determine clear cause–effects relations. There will always be a series of more or less arbitrary trade-offs.

Perhaps the most exciting and currently achievable experimental approach in the world of TFM revolves around combining simultaneous measurement techniques. This could be through merging fluorescent molecular force sensors with classical 2D TFM, to have a better understanding of how forces propagate between the molecular and cellular scales. It could also be through the simultaneous quantification of cellular/molecular forces with signaling cascades, eg. using live phosphorylation (94) or calcium reporters (95, 96), to yield a more complete picture of how force generation and biochemical events are integrated across different scales. Ultimately, studying the mechanobiology of cells in general, but of immune cells and T cells in particular, will be the route to enhancing our understanding of the role of mechanobiology in health and disease (2, 4), and hopefully we will one day be able to translate this wealth of knowledge into next-generation diagnoses and treatments.

## Author contributions

All authors listed have made a substantial, direct, and intellectual contribution to the work and approved it for publication.

## Acknowledgments

The project leading to this publication has received some funding from France 2030, the French Government program managed by the French National Research Agency (ANR-16-CONV-0001) and from Excellence Initiative of Aix-Marseille University - A*MIDEX. Part of this work was also supported by institutional grants from Inserm, CNRS and Aix-Marseille University to the LAI and from CNRS and Aix-Marseille University to the CINAM.

FM was supported as a PhD grant by the European Union’s Horizon 2020 research and innovation programme under the Marie Skłodowska-Curie grant agreement No713750, with the financial support of the Regional Council of Provence- Alpes-Côte d’Azur and with of the A*MIDEX (n° ANR- 11-IDEX-0001-02), funded by the Investissements d’Avenir project funded by the French Government, managed by the French National Research Agency (ANR).

## Conflict of interest

The authors declare that the research was conducted in the absence of any commercial or financial relationships that could be construed as a potential conflict of interest.

## Publisher’s note

All claims expressed in this article are solely those of the authors and do not necessarily represent those of their affiliated organizations, or those of the publisher, the editors and the reviewers. Any product that may be evaluated in this article, or claim that may be made by its manufacturer, is not guaranteed or endorsed by the publisher.
